# Comparative transcriptome analysis of human skeletal muscle in response to cold acclimation and exercise training in human volunteers

**DOI:** 10.1186/s12920-020-00784-z

**Published:** 2020-09-04

**Authors:** Emmani B. M. Nascimento, Roland W. J. Hangelbroek, Guido J. E. J. Hooiveld, Joris Hoeks, Wouter D. Van Marken Lichtenbelt, Matthijs H. C. Hesselink, Patrick Schrauwen, Sander Kersten

**Affiliations:** 1 Department of Nutrition and Movement Sciences, Maastricht Medical Centre, NUTRIM School of Nutrition and Translational Research in Metabolism, Maastricht, The Netherlands; 2 Nutrition, Metabolism and Genomics group, Division of Human Nutrition and Health, Wageningen University, Stippeneng 4, 6708 WE Wageningen, The Netherlands

**Keywords:** Skeletal muscle, Type 2 diabetes, Cold acclimation, Insulin sensitivity, Exercise training

## Abstract

**Background:**

Cold acclimation and exercise training were previously shown to increase peripheral insulin sensitivity in human volunteers with type 2 diabetes. Although cold is a potent activator of brown adipose tissue, the increase in peripheral insulin sensitivity by cold is largely mediated by events occurring in skeletal muscle and at least partly involves GLUT4 translocation, as is also observed for exercise training.

**Methods:**

To investigate if cold acclimation and exercise training overlap in the molecular adaptive response in skeletal muscle, we performed transcriptomics analysis on vastus lateralis muscle collected from human subjects before and after 10 days of cold acclimation, as well as before and after a 12-week exercise training intervention.

**Results:**

Cold acclimation altered the expression of 756 genes (422 up, 334 down, *P* < 0.01), while exercise training altered the expression of 665 genes (444 up, 221 down, P < 0.01). Principal Component Analysis, Venn diagram, similarity analysis and Rank–rank Hypergeometric Overlap all indicated significant overlap between cold acclimation and exercise training in upregulated genes, but not in downregulated genes. Overlapping gene regulation was especially evident for genes and pathways associated with extracellular matrix remodeling. Interestingly, the genes most highly induced by cold acclimation were involved in contraction and in signal transduction between nerve and muscle cells, while no significant changes were observed in genes and pathways related to insulin signaling or glucose metabolism.

**Conclusions:**

Overall, our results indicate that cold acclimation and exercise training have overlapping effects on gene expression in human skeletal muscle, but strikingly these overlapping genes are designated to pathways related to tissue remodeling rather than metabolic pathways.

## Background

A decade ago, we and others demonstrated the presence of brown adipose tissue (BAT) in adult humans and observed that BAT can be activated by cold exposure [1–4]. Since then, there has been a growing interest in the application of cold exposure and/or BAT activation as a strategy to combat obesity and type 2 diabetes (T2D). By virtue of the high expression of the uncoupling protein 1, BAT has the unique property to uncouple mitochondrial respiration from ATP production, leading to heat production [5]. We have previously shown that cold acclimation consisting of daily exposure to 14–15 °C for 6 h for a period of 10 days markedly increased peripheral insulin sensitivity in human volunteers with T2D [6]. However, the detectability of BAT in human volunteers with T2D was very low or even undetectable by FDG-PET/CT, and was only marginally increased after cold acclimation. Strikingly, cold acclimation resulted in a marked increase in GLUT4 translocation to the plasma membrane in skeletal muscle, suggesting that the improvement in insulin sensitivity originates from intrinsic changes in skeletal muscle. Thus far, the molecular pathways triggered by cold acclimation in human skeletal have remained unexplored.

Besides cold acclimation, another way to increase peripheral insulin sensitivity in human subjects is via exercise training [7–9]. Indeed, we and others previously demonstrated that an exercise training intervention in human volunteers both with and without T2D improved peripheral insulin sensitivity, which mainly reflects muscle insulin sensitivity [10, 11], highlighting the usefulness of exercise training in the management of obesity and T2D.

To gain more insight into the molecular pathways activated by cold acclimation and exercise training in skeletal muscle, we performed transcriptomics analysis on vastus lateralis muscle collected from T2D patients before and after 10 days of cold acclimation. In concert, we analyzed the muscle transcriptome in non-diabetic subjects before and after a 12-week exercise training intervention.

## Methods

### Cold acclimation in volunteers with T2D

Experimental procedures of the cold-acclimation study have been previously published [6]. A short summary is provided below. This study included 8 overweight male individuals with T2D (at baseline: age 59 ± 6 years, BMI 29.9 ± 3.1 kg/m^2^; average ± SD). Before the start of the cold acclimation, a muscle biopsy was taken from the vastus lateralis muscle after an overnight fast. During the 10 days of cold acclimation, subjects were exposed to an environmental temperature of 14–15 °C for 10 consecutive days: 2 h on day 1, 4 h on day 2, and 6 h on days 3 through 10. During these times, subjects were dressed in shorts and T-shirt and remained sedentary while staying in the cold room. On day 10, a skeletal muscle biopsy was taken after an overnight fast. Immediately thereafter, a hyper-insulinemic euglycemic clamp was performed as described previously [6, 10].

### Exercise training intervention in healthy volunteers

Experimental procedures of the exercise training study have been previously published [10]. Twenty healthy overweight middle-aged male subjects (at baseline: age 59 ± 4 yr, BMI 29.7 ± 3.6 kg/m^2^, VO_2_ max 28.8 ± 4.3 ml·min^− 1^·kg^− 1^; average ± SD) performed a 12 week combined exercise training intervention. The 20 healthy subjects participated in a larger study and were matched for age, BMI, and maximal oxygen uptake with 18 subjects with T2D. The combined exercise training consisted of two endurance exercise sessions and one resistance exercise session per week (45 min per session). Muscle biopsies were taken following an overnight fast from the vastus lateralis muscle before and 3 days after termination of the training period.

### Muscle biopsies

The Bergström technique with suction was used for collecting the muscle biopsies [12]. All biopsies were taken from a separate incision. Biopsies were separated into aliquots and immediately frozen into liquid nitrogen and stored at − 80 °C for further analysis.

### Microarray analysis

Total RNA was extracted from muscle biopsies using TRIzol reagent (Life Technologies, Bleiswijk, The Netherlands) from 20 healthy volunteers before and after exercise training and from eight volunteers with T2D before and after cold acclimation. Subsequently RNA was purified using the RNeasy Micro kit (Qiagen, Venlo, The Netherlands). RNA integrity was verified with RNA 6000 Nano chips on an Agilent 2100 bioanalyzer (Agilent Technologies, Amsterdam, The Netherlands). Purified RNA (100 ng) was labeled with the Affymetrix WT PLUS reagent kit (Affymetrix, Santa Clara, CA, USA) and hybridized to an Affymetrix Human Gene 1.1 ST array plate (Affymetrix, Santa Clara, CA, USA). Hybridization, washing, and scanning were carried out on an Affymetrix GeneTitan platform according to the manufacturer’s instructions. The array data from two samples in the exercise training study and one sample in the cold acclimation study failed to meet quality control criteria. The corresponding paired samples were also removed from the analysis, leaving complete microarray data for 18 subjects in the exercise training study and 7 subjects in the cold acclimation study. Normalized expression estimates were obtained from the raw intensity values applying the robust multi-array analysis pre-processing algorithm available in the Bioconductor library AffyPLM with default settings [13, 14]. Probe sets were defined and assigned to Entrez IDs using current genome annotation information released by the Genome Reference Consortium (remapped CDF v22) [15]. The *P* values were calculated using an Intensity-Based Moderated T-statistic (IBMT) [16]. Genes were defined as significantly changed when *P* < 0.01. Pathway analysis was carried out using gene set enrichment analysis (GSEA) [17] and EnrichR [18, 19]. For GSEA, genes were ranked based on the IBMT-statistic and subsequently analyzed for over- or underrepresentation in predefined gene sets derived from Gene Ontology, KEGG, National Cancer Institute, PFAM, Biocarta, Reactome and WikiPathways pathway databases. Only gene sets consisting of more than 15 and fewer than 500 genes were taken into account. Statistical significance of GSEA results was determined using 1000 permutations.

The transcriptome datasets are available at NCBI Gene Expression Omnibus under accession numbers GSE53598 and GSE156249.

## Results

**Fig. 1:**
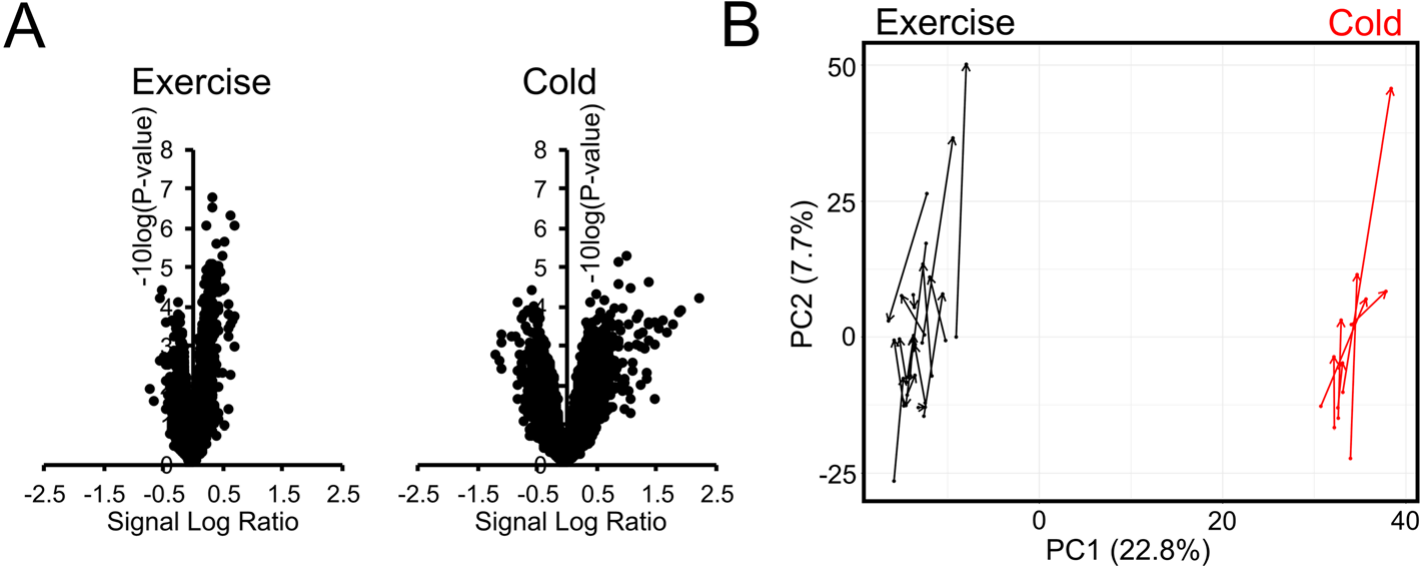
Comparative analysis of cold acclimation and exercise training on the human skeletal muscle transcriptome. **a** Volcano plot in which 2log(fold-change) is plotted against −10log-(*P*-value) for comparison pre-cold acclimation vs post-cold acclimation (left panel), and for the comparison pre-exercise training vs post-exercise training. **b** Principle component analysis of transcriptomics data from human skeletal muscle. The arrows indicate the change from pre-cold acclimation (base of red arrow) to post-cold acclimation (tip of red arrow) and from pre-exercise training (base of black arrow) to post-exercise training (tip of black arrow)

As previously published, in volunteers with T2D (age: 59 ± 6 years; BMI 29.9 ± 3.1 kg/m^2^; average ± SD) cold acclimation significantly increased peripheral insulin sensitivity. Similarly, in healthy volunteers (age: 59 ± 4 years, BMI 29.7 ± 3.6 kg/m^2^; average ± SD), exercise training significantly increased peripheral insulin sensitivity (rate of disappearance of glucose, Rd), as determined by a hyperinsulinemic-euglycemic clamp in combination with stable isotopes [6, 10]. Transcriptomics analysis was performed on muscle biopsies collected before and after 10 days of cold acclimation and 12 weeks of exercise training intervention using Affymetrix microarrays. Cold acclimation altered the expression of 756 genes (422 up, 334 down, *P* < 0.01), while exercise training altered the expression of 665 genes (444 up, 221 down, *P* < 0.01). Volcano plot analysis showed that the signal log ratios for the changes in gene expression induced by cold acclimation were larger than the signal log ratios for the changes induced by exercise (Fig. 1a). As reflected by the height of the volcano, the statistical significance of the gene expression changes was generally higher in response to exercise training than in response to cold acclimation (Fig. 1a). To further analyze the effect of cold acclimation and exercise training on the muscle transcriptome, we performed a principle component analysis (PCA), with the changes in gene expression in each subject visualized by arrows (Fig. 1b). PCA clearly showed a batch effect of the two different experiments in the first dimension, as indicated by the clear separation of the data along the x-axis. Interestingly, all arrows representing the cold acclimation effect pointed upward, suggesting that the overall cold acclimation-induced changes in gene expression were highly similar between subjects. By contrast, the changes in gene expression triggered by exercise were more scattered, suggesting a larger variation in response between subjects. Nevertheless, most arrows were pointing up, indicating similarity in overall gene regulation in skeletal muscle between cold acclimation and exercise training (Fig. 1b).

**Fig. 2:**
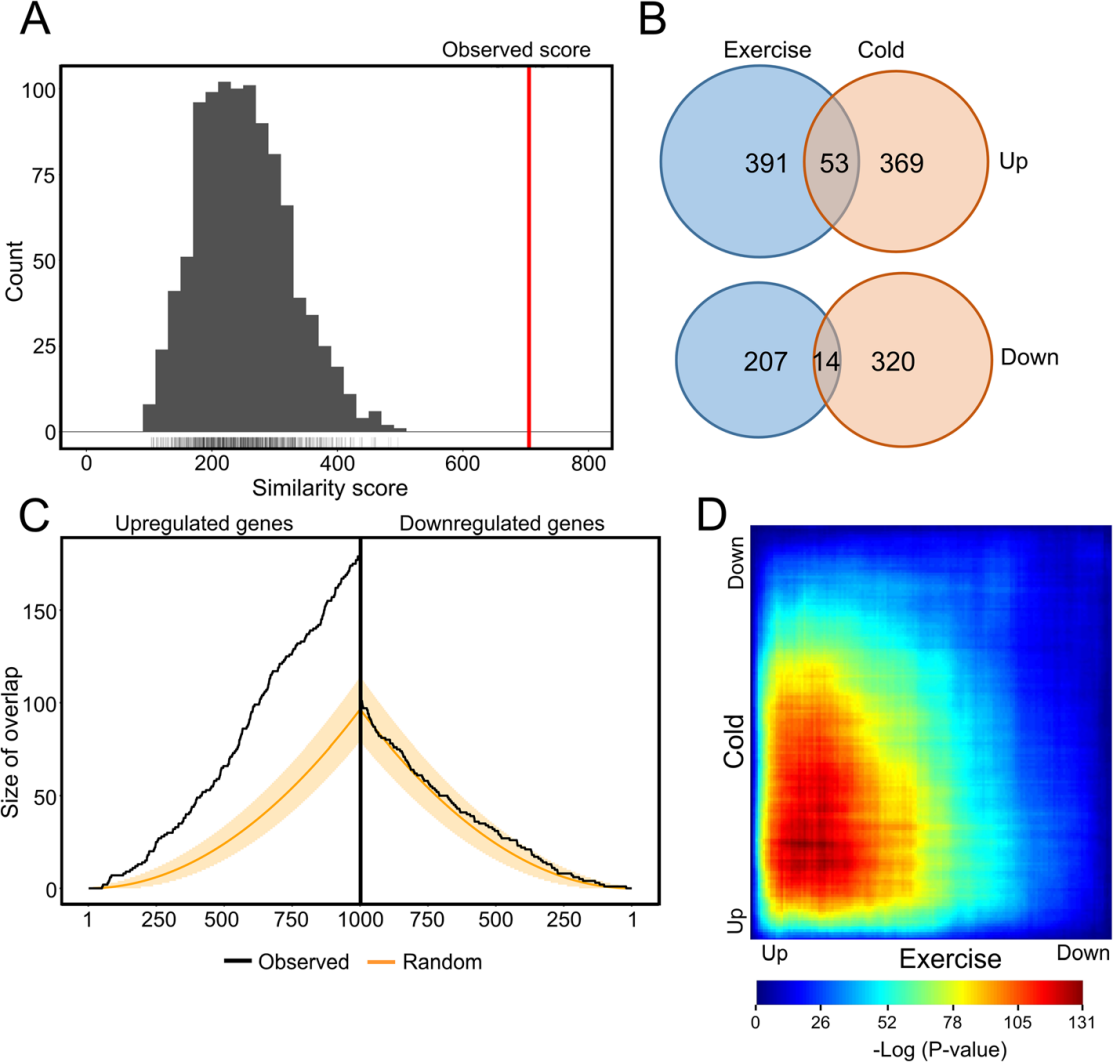
Marked overlap in human skeletal muscle gene expression changes by cold acclimation and exercise training. **a** Score plot showing the distribution of similarity scores based on a random selection of genes from the two datasets. The observed similarity score for the cold and exercise-induced changes in gene expression is depicted by the red line (*P* = 0), showing high significant similarity in gene regulation between the two interventions. The distribution of similarity scores calculated for a random selection of genes in each study is shown in grey. **b** Venn diagrams showing the overlap in genes significantly upregulated (upper diagram) and downregulated (lower diagram) by cold acclimation and exercise training in human skeletal muscle (*P* < 0.01). **c** Overlap plot depicting the size of the overlap for genes upregulated (left) or downregulated (right) by cold acclimation and exercise training. The size of the overlap for randomly selected gene sets is shown by the orange line (beige shading depicts confidence interval). The observed overlap is shown by the black line. **d** Rank–rank Hypergeometric Overlap (RRHO) algorithm was used to determine the statistically significant overlap in gene regulation by cold acclimation and exercise training based on ranked lists of differential gene expression. RRHO analysis determine the level of overlap by stepping through genes ranked by their differential expression in the two interventions, at each point using the hypergeometric distribution to assess the significance of the number of overlapping genes observed

To further explore the similarity in skeletal muscle gene regulation between cold acclimation and exercise, we performed OrderedList analysis [20, 21]. In this analysis, the similarity in the order of genes in two lists is given a score, which is based on the number of shared genes in the top ranked genes. This score is weighted to favor genes at the extremes of the list (i.e. the top up- and downregulated genes). The same scoring method is used on randomly shuffled lists using 1000 permutations to establish a null distribution. The observed similarity score is compared to the scores given when comparing randomly shuffled lists, which can be used to calculate a *p*-value. We used the t-statistic to determine the order of the genes for both the exercise training and cold lists. For the combined analysis of cold acclimation and exercise training, the observed similarity score was much higher than the similarity scores calculated using random shuffling permutations, the distribution of which is shown in grey (*P* < 0.001). This analysis indicates highly significant similarity in gene regulation between the two interventions (Fig. 2a).

Next, we made Venn diagrams to assess the specific overlap in gene regulation between cold acclimation and exercise training. The number of genes upregulated by cold acclimation that were also upregulated by exercise training was 53, corresponding to 12.6%. By contrast, the number and percentage of genes downregulated by cold acclimation that were also downregulated by exercise training was 14, corresponding to 4.2%. Accordingly, the overlap in gene regulation between cold acclimation and exercise training was higher for the upregulated genes than for the downregulated genes (Fig. 2b).

To statistically analyze the overlapping gene regulation, we performed overlap analysis [20, 21] (Fig. 2c). In this analysis, the expected overlap is calculated for any number of top genes (on the x-axis) using a hypergeometric distribution (i.e. over-representation analysis). The orange line and shaded beige area covers the expected overlap under the null hypothesis (95% CI), the black line indicates the observed overlap. Specifically, upregulated genes showed significant overlap between cold acclimation and exercise training as compared to a random selection of genes from both datasets. By contrast, no significant overlap was observed between cold acclimation and exercise training in the downregulated genes, as demonstrated by the overlay with the random plot line (Fig. 2c). To further examine overlapping gene regulation, we used a Rank–rank Hypergeometric Overlap (RRHO) algorithm (Fig. 2)d. This approach tests for significant overlap between two independent gene lists sorted by differential gene expression. Accordingly, we generated a gene list for cold acclimation and a gene list for exercise training, both sorted by t-statistic. This method identifies areas of significant overlap by determining the degree of statistical enrichment using the hypergeometric distribution while sliding across all possible thresholds through the two sorted lists [22], producing a graphical map that visualizes the strength, pattern and bounds of correlation between the two lists. Consistent with the overlap plot analysis, the results showed overlapping gene regulation among the genes upregulated by cold acclimation and exercise training, but not among the downregulated genes (Fig. 2d). Overall, the above analyses demonstrate significant overlap in upregulation of genes by cold acclimation and exercise training, but not downregulation of genes.

**Fig. 3:**
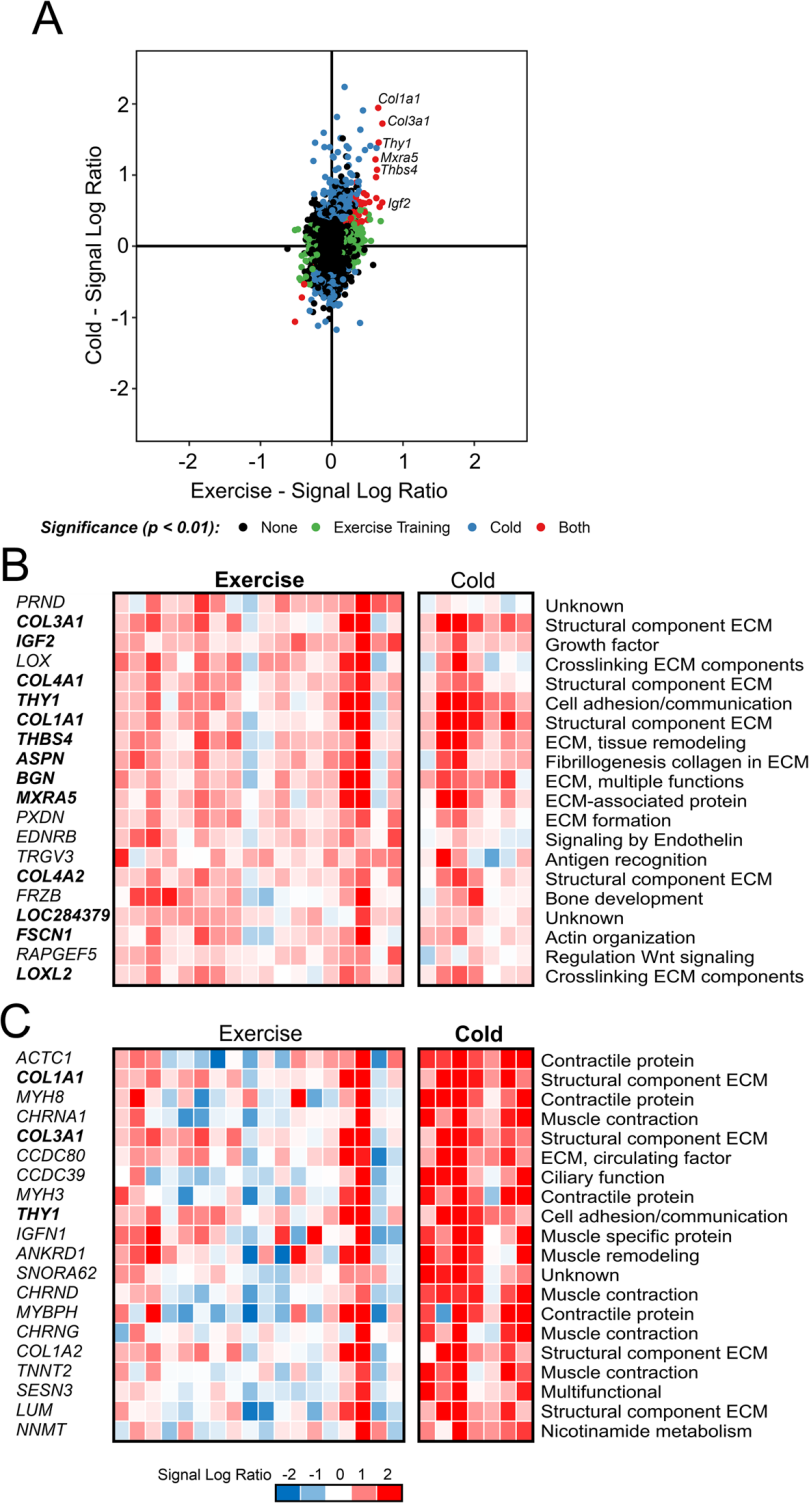
Genes commonly regulated by cold acclimation and exercise training in skeletal muscle from human volunteers. **a** Correlation plot showing gene expression changes in response to cold acclimation (y-axis) vs exercise training (x-axis), expressed as signal log ratio. Genes significantly regulated by exercise training are in green, by cold in blue, and by both interventions in red. **b** The top 20 most highly induced genes by exercise training (*P* < 0.01), ranked according to fold-change. Genes significantly induced by cold acclimation are indicated in bold (*P* < 0.01). **c** The top 20 most highly induced genes by cold acclimation (*P* < 0.01), ranked according to fold-change. Genes significantly induced by exercise training are indicated in bold (*P* < 0.01)

To better understand the overlapping gene regulation, we visualized gene expression changes in a scatter plot (Fig. 3a). The elliptical shape of the scatter indicates the overall similarity in gene regulation by cold acclimation and exercise training, thereby underscoring the previous analyses. Genes significantly regulated by both cold acclimation and exercise training were mainly found in the first quadrant, reflecting upregulation (Fig. 3a). These genes include the collagens *COL1A1* and *COL3A1*, the adhesive glycoprotein *THBS4*, insulin-like growth factor *IGF2*, T-cell antigen *THY1* and matrix remodeling-associated protein 5 *MXRA5*. To further zoom in on the specific genes regulated by the two interventions, we visualized in each of the volunteers the gene expression changes of the top 20 most highly induced genes in each of the two interventions (Fig. 3b, c). Overall, the gene expression changes were variable between volunteers, which is typical for human interventions. Interestingly, the majority of the top 20 genes induced by exercise training was also induced by cold acclimation, including many genes typically induced by exercise such as *COL3A1*, *COL4A1*, *COL1A1*, *THBS4, MXRA5* (Fig. 3b). These genes are part of the extracellular matrix and reflect the extracellular matrix structural remodeling that is characteristic of the repeated exercise bout effect [23]. Accordingly, these data suggest that similar to exercise training, cold acclimation also causes transcriptional remodeling of the extracellular matrix.

By contrast, a much smaller portion of the top 20 cold acclimation-induced genes was also induced by exercise training (Fig. 3c). Many of the most highly induced genes by cold acclimation encode proteins involved in muscle contraction. These genes, which include contractile genes such as *ACTC1*, *MYH8*, and *MYBPH*, as well as genes involved in signaling between nerve and muscle cells such as *CHRNA1*, *CHRND*, and *CHRNG*, are not consistently induced by exercise training (Fig. 3c).

**Fig. 4:**
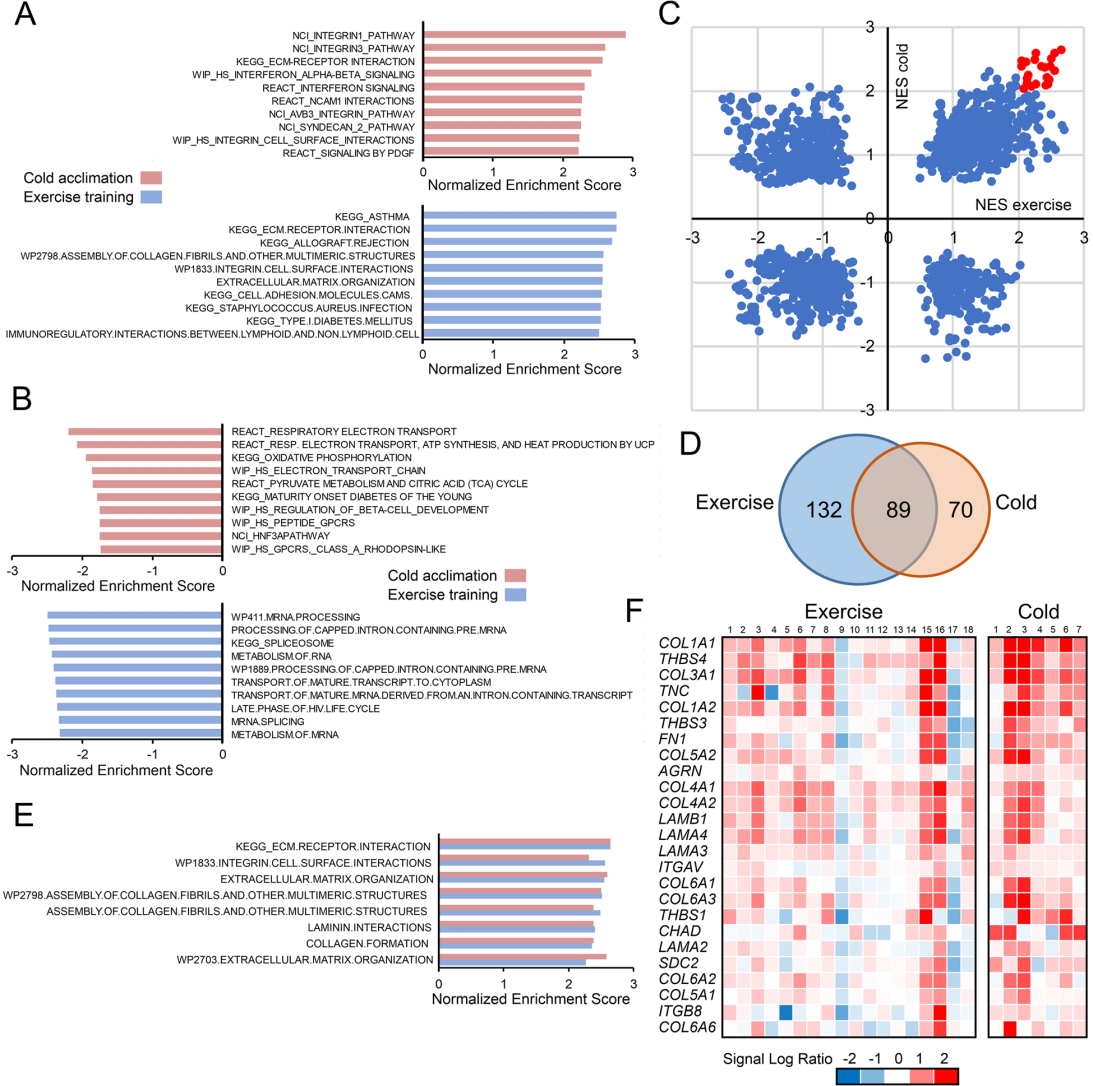
Effects of cold acclimation and exercise training on gene expression at the pathway level. **a** Top 10 positively enriched (upregulated) gene sets in human skeletal muscle in response to cold acclimation (red bars), and exercise training (blue bars) according to gene set enrichment analysis. **b** Top 10 negatively enriched (upregulated) gene sets in human skeletal muscle in response to cold acclimation (red bars), and exercise training (blue bars) according to gene set enrichment analysis. **c** Correlation plot of Normalized Enrichment Scores (NES) derived from GSEA for the comparison pre-cold vs post-cold (y-axis) and the comparison pre-exercise vs post-exercise (x-axis). Gene sets with NES values above 2 in both interventions are indicated in red. **d** Venn diagram of significantly upregulated gene sets (q < 0.05) in human skeletal muscle in response to exercise training and cold acclimation. **e** Gene sets meeting statistical significance q < 0.0001 for the comparison pre-cold vs post-cold (red) and the comparison pre-exercise vs post-exercise (blue). **f** Heatmap showing individual gene expression changes of the 25 most highly induced genes by cold that are part of the gene set KEGG_ECM.RECEPTOR.INTERACTIONS. Red indicates upregulated, blue indicates downregulated

To further elucidate the biological pathways activated by cold acclimation, we performed gene set enrichment analysis (GSEA). GSEA uses a priori gene sets and quantifies the positive or negative enrichment of genes within a particular gene set in a gene list sorted by differential gene expression. Consistent with the top 20 genes presented above, gene sets related to extracellular matrix (ECM) and ECM signaling were highly enriched among the cold acclimation-induced genes (Fig. 4a). A similar phenomenon was observed for genes upregulated by exercise training, showing strong enrichment for ECM pathways (Fig. 4a).

Interestingly, gene sets related to the respiratory chain and oxidative phosphorylation were enriched among the cold acclimation-repressed genes, suggesting downregulation of oxidative phosphorylation (Fig. 4b). These gene sets did not appear in the analysis of genes downregulated by exercise training, suggesting the effect is specific to cold acclimation (Fig. 4b). Scatter plot analysis of all gene sets based on enrichment scores showed considerable similarity in upregulation of gene sets by cold acclimation and exercise training, as reflected by the elliptical shape of the scatter in the first quadrant (Fig. 4c), suggesting that cold acclimation and exercise training upregulated similar pathways. The gene sets with positive enrichment scores > 2 in both interventions are shown in red. Most of these gene sets are related to ECM. This elliptical shape was not observed in the third quadrant, suggesting no similarity in downregulation of gene sets by cold acclimation and exercise training (Fig. 4c).

Additionally, using a cut-off of q < 0.05 for statistical significance of positively enriched gene sets, a Venn diagram showed major overlap between gene sets upregulated by cold acclimation and gene sets upregulated by exercise, again underscoring the similarity in gene upregulation between the two interventions (Fig. 4d). When raising the statistical significance bar even higher to a very stringent q < 0.0001, 8 gene sets were found overlapping between the cold acclimation and exercise training interventions, all of which were related to the ECM (Fig. 4e). The individual expression changes of genes in the gene set ECM.RECEPTOR.INTERACTIONS are visualized in Fig. 4f. These data show that in all individuals except two, cold acclimation and exercise training consistently induced the expression of numerous genes within this gene set.

**Fig. 5:**
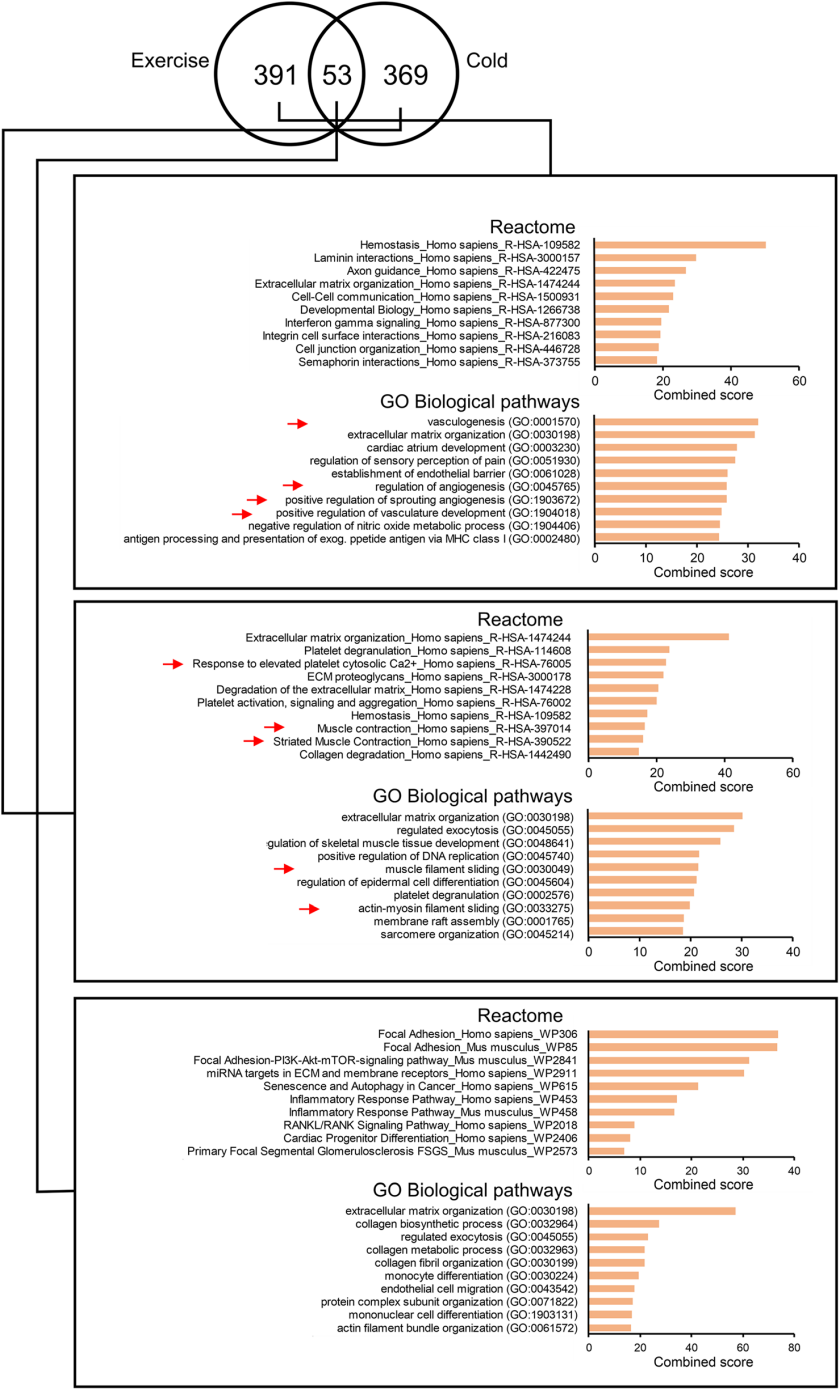
Pathways specifically induced by cold acclimation and exercise training in human skeletal muscle. On the basis of the Venn diagram shown in Fig. 2b for genes significantly upregulated by cold acclimation and/or exercise training (*P* < 0.01), EnrichR analysis was carried out on the set of genes exclusively induced by exercise training (left), cold acclimation (right) or combined. The pathways highlighted with a red arrow are of particular interest

As an alternative approach to examine the overlap in upregulation of biological pathways by cold acclimation and exercise training, we performed pathway analysis using EnrichR (Fig. 5). In contrast to GSEA, EnrichR starts with a list of differentially expressed genes and determines the enrichment of these genes on the basis of gene ontology or predefined pathways. EnrichR was carried out using the three groups of genes shown in Fig. 2b, representing cold acclimation-specific upregulation, exercise training-specific upregulation, and shared upregulation. Interestingly, many of the most significant exercise training-specific GO Biological Pathways were related to angiogenesis. Moreover, pathways related to intercellular interactions were clearly overrepresented among the exercise training-specific Reactome pathways (Fig. 5). In support of the cold acclimation-specific induction of contractile proteins, pathways connected to muscle contraction were overrepresented among the cold acclimation-specific Reactome and GO pathways. In addition, several of the cold acclimation-specific pathways were related to ECM. Importantly, pathways connected to ECM and ECM signaling also featured prominently among the pathways shared by cold acclimation and exercise training, further suggesting similarity in upregulation of biological pathways between cold acclimation and exercise training. Collectively, these analyses showed that pathways related to muscle contraction were specifically induced by cold acclimation, whereas pathways related to ECM were induced by both cold acclimation and exercise training.

## Discussion

In this paper, we compare the transcriptomic profiles of human skeletal muscle in response to repeated mild cold exposure versus exercise training. Collectively, the different types of analyses provide strong evidence for significant overlap between cold acclimation and exercise training with respect to upregulation of gene expression in skeletal muscle, but not downregulation of gene expression. The overlap in gene regulation is particularly prominent for genes and pathways connected to the extracellular matrix. Surprisingly, several of the genes most highly induced by cold acclimation are involved in contraction and in signaling between nerve and muscle cells. These results may suggest that the effects of cold acclimation on muscle insulin sensitivity are likely caused by muscle contraction/shivering during the acclimation period, thereby downplaying the possible contribution of non-shivering thermogenesis to the reported effects of cold exposure on insulin sensitivity.

Remarkably, the fold changes in gene expression in response to cold acclimation are quite pronounced. The genes most strongly induced by cold acclimation include genes involved in contraction and signaling between nerve and muscle cells, as well as genes involved in the extracellular matrix, such as various collagens. The functional rationale behind these changes remains elusive. It is conceivable that the changes in genes involved in contraction and nerve-muscle signaling are an adaptive response aimed at enhancing the intensity and efficiency of shivering. The alterations in genes involved in the extracellular matrix may reflect a change in structural organization of skeletal muscle following repeated cold exposure, the purpose of which remains unclear. Overall, the marked changes in gene expression indicate that even mild, intermittent cold exposure has a major impact on skeletal muscle in humans.

We previously published that cold acclimation increased peripheral insulin sensitivity, likely by increasing GLUT4 translocation in skeletal muscle [6]. Interestingly, the enhanced GLUT4 translocation was already observed in the overnight fasted, non-insulin stimulated state. Although we previously did not see an effect of cold acclimation on AMPK activation and subjects did not report visual shivering, the data herein suggest that the improved insulin sensitivity likely is the result of mild shivering or muscle tension. Our findings therefore highlight the importance of further studying the potential of mild shivering to improve metabolic health in humans and also raise the question whether cold-induced non-shivering thermogenesis alone is sufficient to elicit metabolic changes.

Several studies have previously examined the effects of exercise training on the skeletal muscle transcriptome in humans [24–27]. Despite differences in the type of exercise and exercise duration, all studies find that exercise changes the expression of numerous genes involved in the extracellular matrix, especially different collagen-encoding genes, supporting the notion that exercise training causes extracellular matrix structural remodeling [23]. Our muscle transcriptome analysis of subjects who followed a mixed exercise protocol—combining endurance and resistance exercise—are consistent with this notion. Interestingly, exercise training did not lead to marked changes in metabolic genes, suggesting that the adaptive changes in oxidative metabolism following exercise training are largely accounted for by other regulatory mechanisms.

The analysis presented here has several limitations. First of all, the number of subjects in each intervention was different. Specifically, the cold acclimation intervention had fewer subjects and accordingly less power, which impacted the statistical significance of the gene expression changes. Second, the microarrays for the two interventions were not run at the same time, creating a batch effect in the combined analysis (Fig. 1b). Third, the timing of the collection of the muscle biopsies was different due to the study design. Muscle biopsies were collected 24 h after the last bout of cold acclimation, whereas exercise trained muscle biopsies were collected 48–72 h after the last exercise bout. The difference in timing may explain the higher fold changes in gene expression in response to cold acclimation compared to exercise training. Fourth, the duration of the interventions was different, which perhaps may have influenced the results. Fifth, the two interventions involved volunteers with or without T2D. By contrast, all experiments were performed at the same facility. Furthermore, we used the same types of microarrays for the two studies, which were run by the same technician and in the same laboratory.

## Conclusions

Cold acclimation and exercise training increase peripheral insulin sensitivity in volunteers with T2D. Whole genome expression analysis of skeletal muscle suggests that cold acclimation is accompanied by remodeling of the extracellular matrix, which is also seen following exercise training, and by contraction and signaling between nerve and muscle cells. Our results suggest that mild shivering is a potential future strategy to increase peripheral insulin sensitivity in volunteers with T2D.

## Data Availability

The datasets generated and/or analysed during the current study are available in the Gene Expression Omnibus repository (GSE53598 and GSE156249).

## Abbreviations

T2D: Type 2 diabetes
BAT: Brown adipose tissue
BMI: Body mass index
GSEA: Gene set enrichment analysis
PCA: Principle component analysis
RRHO: Rank-Rank Hypergeometric Overlap
ECM: Extra-cellular matrix
